# Deconstructing Kinsey's Scale: Digit Ratio Correlates Negatively with Gynephilia and Positively with Androphilia in Both Sexes

**DOI:** 10.1007/s12110-026-09518-z

**Published:** 2026-04-15

**Authors:** Oliver C. Schultheiss, David Gebhardt, Elisabeth Meier, Sophie Steck, Monika Wulf, Paul Compensis, Martin G. Köllner

**Affiliations:** 1 Friedrich-Alexander University, Erlangen, Germany; 2 SRH University of Applied Sciences Heidelberg, Fürth, Germany

**Keywords:** Digit ratio, Ulna-fibula ratio, Sexual orientation, Androphilia, Gynephilia, Organizing hormone effects

## Abstract

Sexual orientation may be shaped, in part, by organizing effects of gonadal steroids during development. However, the interpretation of associations between sexual orientation and markers of such organizing effects may be hampered by the use of Kinsey-type scales of hetero-/homosexuality, because they assess sexual orientation from the individual’s (i.e., egocentric) perspective and inherently represent a difference score between attraction to women versus attraction to men, with unclear meaning of medium scores. Using illustrative data from three studies, we show that a clearer and more consistent pattern emerges once one uses measures of sexual orientation that assess sexual attraction from the (allocentric) perspective of one’s object of attraction; that is, as attraction to women (gynephilia) and to men (androphilia). We find that, in both men and women, gynephilia is negatively, and androphilia positively, associated with digit ratio, a measure of the relative prenatal exposure to testosterone versus estradiol. This suggests that relatively more exposure to testosterone predisposes individuals to be more attracted to women and less to men and vice versa for relatively more exposure to estradiol. We also find that ulna-to-fibula ratio, a putative marker of pubertal steroid effects, is positively associated with gynephilia, particularly in men, suggesting that relatively more testosterone exposure during puberty may be another source of individuals’ sexual attraction to women. When we constructed a Kinsey-type measure of homo-/heterosexuality from androphilia and gynephilia scales, our findings for digit ratio replicated those reported in the literature for generic Kinsey-type scales. But we argue that the findings we obtained with allocentric gynephilia and androphilia measures are more straightforward to interpret.

**Supplementary Information:**

The online version contains supplementary material available at 10.1007/s12110-026-09518-z.

Sexual orientation, that is, the extent to which one feels sexually attracted to one’s own sex, the other sex, or both, has profound consequences for sexual behavior and partner preferences, but also societal practices and political discourse (Bailey et al., 2016). Understanding the origins of sexual orientation therefore remains an important goal, and scientific explanations range from social learning (Ågmo, 2007) to immunological (Bogaert & Skorska, 2011) to hormonal accounts (Balthazart, 2011; Swift-Gallant et al., 2023). It is the latter approach we will focus on here, with an emphasis on morphological markers of organizing hormone effects, such as digit ratio (or 2D:4D; Manning, 2002), for which there is already substantial evidence for an association with sexual attraction (e.g., Swift-Gallant et al., 2025), and ulna-fibula-ratio (or UFR; Köllner and Bleck 2020a, b), for which our study is exploratory. What many of the studies conducted under the auspices of different accounts of sexual orientation (see Sell, 1997, for a review of sexual orientation measures) have in common is that they employed measures that can be traced back to the pioneering work of Kinsey and colleagues (1948) and used scales to classify individuals into varying degrees of hetero- and homosexuality, with scale endpoints representing exclusive heterosexual and exclusive homosexual orientation. Using data collected in three different studies and that included markers of prenatal (digit ratio) and pubertal hormone exposure (UFR) as an illustration, we argue in the present paper that such a measurement approach may obscure more than it reveals.

## The Kinsey Scale of Sexual Orientation

Kinsey and colleagues (1948) employed a seven-point scale to capture variations in sexual orientation ranging from *exclusively heterosexual* (0) through *equally heterosexual and homosexual* (3) to *exclusively homosexual* (6). They used the scale to classify the individuals they interviewed and did this based on the advanced understanding (for their time) that homo- and heterosexuality do not represent separate classes but endpoints of a continuum of sexual orientation, with many people falling in between the scale endpoints. At its core, however, the Kinsey scale and its various adaptations and descendants (e.g., Sell, 1997) is plagued by two main problems: it represents an *egocentric view* of sexual orientation and it is inherently a *difference score*.

The Kinsey scale is egocentric in the sense that it operationalizes sexual orientation as viewed from the person to be assessed. Regardless of whether it is a woman or a man who is being measured, homo- and heterosexuality appear to mean the same thing; that is, whether one is attracted to the other sex or one’s own sex. But one could also take an allocentric assessment perspective and anchor sexual orientation not in one’s own attraction to the same or the other sex but in the degree of sexual attraction that women and men exert on oneself (e.g., Hale et al., 2019). This aspect of sexual orientation is better captured by the concepts of gynephilia and androphilia; that is, the degree to which one feels sexually attracted to women and/or men, respectively (e.g., Blanchard, 1985). With this approach, both men and women can be attracted to other men (androphilia) or women (gynephilia) to varying degrees, and whether this is then classified in a secondary step as a hetero- or homosexual attraction depends on the match between subject sex and attraction object sex.^1^

Kinsey-type scales also suffer from the fact that they are truly difference scales, because the scale score inherently represents the difference between how much one feels attracted by a member of the other sex versus by a member of one’s own sex (Zietsch & Sidari, 2020). However, the two underlying dimensions of attraction – attraction to one’s own sex and attraction to the other sex – are not mutually exclusive. In principle, one could be high in one, both, or neither. But the Kinsey scale represents only the exclusive-orientation endpoints faithfully; non-exclusive admixtures between 0 and 6 present problems. For instance, the scale midpoint of 3 could be generated by matching high or low strengths on the two underlying dimensions, rendering the meaning of a score of 3 ambiguous with regard to whether it signifies true, strong attraction to both sexes (i.e., bisexuality) or no attraction to either sex (i.e., asexuality). Kinsey and colleagues (1948) were aware of this interpretational conundrum and therefore allowed assignment of an additional X score if a person was asexual. But this begs the question: Why add a second variable to disambiguate the meaning of the difference score of two variables if a more transparent approach using two independent dimensions of sexual attraction could have been used from the get-go? Note that this problem pertains to some extent not only to the midpoint of the scale but also to other scale points between the extremes, because it is never clear to what extent the relative difference between hetero- and homosexuality is driven by attraction to one sex or the other.

What’s more: In a worst-case scenario a difference score may obscure the specific and strong contribution of one measure to effects captured with the difference score, while the other measure may only add statistical noise to it. Nevertheless, findings obtained with the difference score are then interpreted, implicitly or explicitly, as if they reflect equal, and equally valid, contributions by each of the two measures.

Research with measures that inherently represent difference scores has led to problems in other lines of inquiry, too. Consider the case of achievement motivation research. Atkinson (1957) advocated the conceptual separation of an approach-oriented component of the achievement motive, called hope of success, and an avoidance-oriented component, called fear of failure. In his research program (e.g., Atkinson & Feather, 1966), he assessed the former through content-coding of picture stories and the latter with a test anxiety questionnaire. After standardizing the two measures, he then created a difference score from them, called resultant achievement motivation, and used it to test hypotheses about hope of success, fear of failure, and their interplay with motivational incentives. This approach was later criticized (Covington & Roberts, 1994; Schultheiss & Brunstein, 2005) because (a) medium scores on the resultant motivation measure were ambiguous with regard to what they represented, (b) the self-report measure of fear of failure was incommensurable with the picture-story measure of hope of success and the two should never have been squeezed into one difference score, and (c) it was now impossible to decide to what extent results reported in the published literature were due to the test-anxiety-measure component of the resultant achievement motivation measure or to the picture-story-measure component. The harvest of a large number of studies on achievement motivation was thus compromised by Atkinson’s initial decision to prefer the resultant achievement motivation score over the use of two independent measure scores capturing hope of success and fear of failure separately.

Research on sexual motivation could have taken a similar turn – but has not. Similar to the distinction between hope of success and fear of failure in achievement motivation research, the Dual Control Model of Sexual Response (Janssen & Bancroft, 2023) distinguishes between approach-oriented sexual excitation and avoidance-oriented sexual inhibition, which independently and jointly determine behavioral outcomes such as sexual risk taking and activity (e.g., Janssen & Bancroft, 2023; Toates, 2014). However, Janssen and Bancroft (2023) have stressed the importance of keeping measures of these motivational orientations separate and modeling them as main and interaction effects in data analyses to properly understand their independent and conjoint effects on behavior. Luckily for Dual Control Model, researchers have mostly refrained from turning measures of sexual excitation and inhibition into difference scores and instead examined their separate and interactive predictive effects. This has ensured that research findings from this approach keeps the specific contribution of each aspect of sexual motivation separate and transparent.

Returning to Kinsey-type scales of sexual attraction, let us emphasize that such measures are not explicit difference scores in the same manner as Atkinson’s resultant achievement score was. However, they represent differences between two latent dimensions *implicitly* and are thus vulnerable to the same criticisms that have been articulated in other difference-score literatures. In the present research, we aim to resolve the egocentrism and the difference-score problems of Kinsey-type scales by employing separate measures of androphilia and gynephilia to capture women’s and men’s sexual orientation in a more nuanced manner.

### Hormonal Factors Associated with Sexual Orientation

There is growing evidence for an association between measures of organizing hormone effects and sexual orientation (Balthazart, 2011). The steroid hormones testosterone and estradiol exert such effects during sensitive developmental phases, particularly during gestation and later during puberty (Breedlove, 2017; Sisk & Zehr, 2005). They are organizing in the sense that they shape the development of the body in the female or male direction. But they also influence other features of the body, such as bone growth patterns, and of the brain, eliciting individual and sexual differences in cognition, emotion/motivation, and behavior (e.g., Schulz & Sisk, 2016). Bone growth patterns that sensitively reflect the relative exposure to testosterone (which facilitates/prolongs growth) and estradiol (which ends/shortens growth) can serve as bodily markers of the organizing effects that gonadal steroid hormones have had on the brain (for review, Köllner et al., 2019).

One well-established marker of prenatal testosterone and estradiol exposure is digit ratio, the ratio of the second to the fourth digit (Manning, 2002). Evidence from studies conducted with animal models shows that digit ratio is sensitive to the relative exposure in the first third of gestation to testosterone (which decreases the ratio) relative to estradiol (which increases the ratio; Zheng & Cohn, 2011). Consistent with the sex difference in exposure to testosterone and estradiol during pregnancy, women have higher ratio scores than men (e.g., Butovskaya et al., 2021). Corroborating research on mice (Zheng & Cohn, 2011), this sex difference is already established by the end of the first trimester of human pregnancies (Malas et al., 2006).

An association between digit ratio and sexual orientation emerged in early studies on this marker of organizing hormone effects (e.g., Manning, 2002; Manning et al., 2007; van Anders & Hampson, 2005) and has since then been confirmed meta-analytically (for women only: Grimbos et al., 2010; for both women and men: Swift-Gallant et al., 2025). Using Kinsey-type, egocentric measures of sexual orientation, these studies found replicable evidence for more male-typical digit ratios in homosexual women and more female-typical digit ratios in homosexual men, as compared to heterosexual women and men, respectively. Bisexual women and men did not differ from heterosexual women and men. Because attraction to individuals of one’s own versus the other sex was not measured separately, however, the null finding for bisexuality may be due to the problems associated with difference measures described previously. More generally, because of their use of egocentric, unidimensional measures of sexual orientation, the studies entering these meta-analyses do not help to resolve the question of whether early prenatal exposure to variations in testosterone and estradiol affects later sexual attraction to women, to men, or both.

### The Present Research

Our goals in the present research were fourfold. First, we wanted to employ allocentric measures of sexual orientation – namely, assessments of sexual attraction to women (gynephilia) and to men (androphilia) – to examine to what extent each measure is associated with morphometric markers of gonadal steroid exposure. Second, we wanted to explore if such relationships differ by natal sex or are similar in women and men. Third, by constructing a Kinsey-type scale of homo-/heterosexuality from measures of gynephilia and androphilia, we wanted to compare our findings both directly to previous studies which have used Kinsey-tape scales, but also to illustrate the advantages of allocentric measures of sexual orientation over an egocentric measure in terms of transparency of the findings and interpretational ease.

Fourth, we wanted to extend our analysis of organizing hormone effects on sexual orientation to a putative marker of pubertal hormone exposure, the ulna-fibula ratio (UFR; Köllner and Bleck 2020a, b; Köllner et al. 2022a, b). Köllner and Bleck (2020a, b) summarize literature that shows that sex differences in long bone growth emerge during puberty. They argue that while such differences influence overall skeletal height, there are also more subtle differences that go beyond overall height, such as sex-specific differences in ulna and fibula lengths. Köllner and colleagues showed in a series of studies that the ratio of the length of these bones is (a) sex-dimorphic, (b) its variations are associated with implicit motivational striving, and (c) in young adults it is associated with salivary testosterone and estradiol, which holds both for basal levels and for hormonal reactions to a dominance contest (Köllner and Bleck 2020a, b; Köllner et al. 2022a, b). Given recent advances in research on how gonadal steroids affect the developing brain during puberty (e.g., Beking et al., 2018; Nguyen, 2018), we wanted to explore whether variations in pubertal hormone exposure, approximated by UFR, are related to sexual orientation, too.

Although the three studies for whose combined data we report findings here were not conducted with our present research question in mind and relevant hypotheses were therefore not preregistered, the following hypotheses can be readily deduced based on previous research and meta-analyses of sexual orientation and digit ratio:

First, women and men differ in gynephilia and androphilia, with women being on average higher than men on androphilia and men being on average higher than women on gynephilia.

Second, women and men differ on bone-growth markers of exposure to testosterone and estradiol, with women having higher scores on digit ratio than men and men having higher scores on UFR than women.

Third, within each sex a more female-typical (i.e., higher) digit ratio is associated with less gynephilia. Conversely, a more male-typical (i.e., lower) digit ratio is associated with more gynephilia. Such a finding would be consistent with a lower digit ratio being associated with a more homosexual orientation in women but a more heterosexual orientation in men.

Fourth, within each sex a higher digit ratio (as more commonly found in women) is associated with more androphilia. Conversely, a lower digit ratio (as more commonly found in men) is associated with less androphilia. Such a finding would be consistent with a higher digit ratio being associated with a more homosexual orientation in men but a more heterosexual orientation in women.

Fifth, for UFR our analyses were exploratory. Although it could be argued that more of the same hormones during puberty that already shaped some aspects of sexual orientation prenatally should lead to more of the same associations with sexual orientation, there is also evidence that perinatal and pubertal steroids can have opposite effects on the same psychological function (see, for instance, Beking et al., 2018). We therefore expected to see an association between UFR and sexual orientation measures, but refrained from specifying a direction.

## Method

### Participants

We combined data from three studies conducted with individuals recruited at Friedrich-Alexander University, Erlangen, Germany, between December 2023 and June 2025 and in which we had collected digit ratio, UFR, and sexual attraction measures in the same manner. Studies 1 and 2 were conducted in a research project focusing on inhibition effects on emotion and behavior; Study 3 examined Pavlovian conditioning effects in the context of thematic apperception. In all studies, we retained for further analyses only those participants who (a) had completed a biographical questionnaire with questions pertaining to their natal sex and their gynephilia and androphilia and (b) for which we either had digit ratio or UFR data (or both).

In Study 1, 90 participants were originally tested. Of those, 89 participants fulfilled the retention criteria, yielding a sample of 68 women and 21 men with a mean age of 22.11 years (*SD* = 4.93), for which we had data available for either UFR (*N* = 89) or digit ratio (*N* = 84).

In Study 2, 171 participants were originally tested. Of those, 163 participants fulfilled the retention criteria, yielding a sample of 123 women and 40 men with a mean age of 22.79 years (*SD* = 3.85), for which we had data available for either UFR (*N* = 161) or digit ratio (*N* = 115).

In Study 3, 120 participants were originally tested and also retained according to our criteria. The sample was comprised of 67 women and 53 men with a mean age of 25.38 years (*SD* = 10.07), for which we had data available for either UFR (*N* = 119) or digit ratio (*N* = 116).

The combined sample thus consisted of 372 participants (258 women, 114 men) with a mean age of 23.47 years (*SD* = 6.85)^2^. Total *N*s for analyses involving digit ratio and UFR are listed per analysis in Tables 1, 2, 3, 4, 5 and 6.

**Table 1:** Descriptive statistics and sex differences for all measures

Variable	Women			Men				
	*n*	*M*	*SD*	*n*	*M*	*SD*	*d*	*95% CI*
Digit ratio	216	0.977	0.031	99	0.962	0.034	0.45***	0.21; 0.69
UFR	255	0.671	0.036	114	0.683	0.043	0.30**	0.08; 0.52
Gynephilia	258	2.03	1.11	114	4.57	0.92	2.40***	2.11; 2.67
Androphilia	258	4.08	0.96	114	1.60	1.11	2.47***	2.19; 2.75
Kinsey	258	1.95	1.59	114	1.03	1.78	0.56***	0.34; 0.79
Dir. attraction	258	2.05	1.59	114	−2.97	1.78	3.04***	2.73; 3.36

**p <.01, ***p <.001

**Table 2:** Pearson correlations (*n*), partialed for study

	Total sample							Men (above diagonal), women (below diagonal)					
Variable		1	2	3	4	5	6	2	3	4	5	6	7
1. Sex		—											
2. Gynephilia		− 0.73***	—					—	− 0.57***	− 0.86***	− 0.86***	− 0.15	0.19*
		(372)							(114)	(114)	(114)	(99)	(114)
3. Androphilia		0.75***	− 0.68***	—				− 0.18**	—	0.91***	0.91***	0.21*	− 0.16
		(372)	(372)					(258)		(114)	(114)	(99)	(114)
4. Kinsey		0.24***	0.04	0.10	—			0.81***	− 0.72***	—	−1.00	0.21*	− 0.20*
		(372)	(372)	(372)				(258)	(258)		(114)	(99)	(114)
5. Directional Attraction		0.81***	− 0.92***	0.92***	0.03	—		− 0.81***	0.72***	1.00	—	0.21*	− 0.20*
		(372)	(372)	(372)	(372)			(258)	(258)	(258)		(99)	(114)
6. Digit ratio		0.20***	− 0.26***	0.24***	0.01	0.27***	—	− 0.19**	0.08	− 0.18**	0.18**	—	− 0.19
		(315)	(315)	(315)	(315)	(315)		(216)	(216)	(216)	(216)		(99)
7. Ulna-fibula		− 0.17***	0.20***	− 0.15**	− 0.11*	− 0.19***	− 0.08	0.07	0.06	0.01	− 0.01	0.03	—
Ratio		(369)	(369)	(369)	(369)	(369)	(312)	(255)	(255)	(255)	(255)	(213)	

Contrast dummy variables were used for partialing. Sex is coded male = 0, female = 1

*p <.05; **p <.01; ***p <.001

**Table 3:** Hierarchical regression results for directional attraction, predicted from digit ratio (*N* = 315)

Variable	Model 1			Model 2			Model 3			Model 4		
	*B (SE)*	*95% CI*	*β*	*B (SE)*	*95% CI*	*β*	*B (SE)*	*95% CI*	*β*	*B (SE)*	*95% CI*	*β*
Constant	0.86** (0.31)	0.25; 1.46		−12.31*** (2.82)	−17.85; −6.77		−13.08** (4.70)	−22.33; −3.83		−17.25 (11.55)	−39.98; 5.58	
SC1	−0.01 (0.40)	−0.81; 0.78	−0.00	−0.05 (0.24)	−0.52; 0.41	−0.01	−0.05 (0.24)	−0.52; 0.41	−0.01	10.72 (16.62)	−21.99; 43.42	1.81
SC2	−0.99* (0.40)	−1.78; −0.19	−0.17	0.04 (0.24)	−0.43; 0.52	0.01	0.04 (0.24)	−0.43; 0.51	0.01	3.10 (12.91)	−22.31; 28.51	0.53
Sex				4.85*** (0.21)	4.44; 5.26	0.79	6.05 (5.91)	−5.58; 17.68	0.99	7.88 (13.50)	−18.69; 34.45	1.29
Digit ratio (DR)				9.76*** (2.92)	4.01; 15.50	0.11	10.55* (4.89)	0.34; 20.17	0.12	14.96 (12.04)	−8.72; 38.64	0.17
Sex x DR							−1.24 (6.11)	−13.27; 10.78	−0.20	−3.27 (14.01)	−30.84; 24.29	−0.52
SC1 x Sex										−6.57 (16.05)	−38.16; 25.01	−0.93
SC2 x Sex										−3.06 (18.85)	−40.15; 34.03	−0.48
SC1 x DR										−3.39 (13.44)	−29.84; 23.06	−0.56
SC2 x DR										−11.08 (17.33)	−45.18; 23.02	−1.83
SC1 x Sex x DR										3.15 (19.57)	−35.36; 41.66	0.49
SC2 x Sex x DR										7.12 (16.62)	−25.58; 39.82	0.98
*R²*	0.028			0.670			0.670			0.674		
*ΔR²*	0.028*			0.642***			0.000			0.004		
*AIC*	1551.73			1215.29			1217.25			1225.63		
*BIC*	1566.74			1237.81			1243.52			1274.41		

*SC1* study contrast 1 (Study 2 [1] versus Study 1 [0]), *SC2* study contrast 2 (Study 3 [1] versus Study 1 [0]), *DR* digit ratio, *AIC* Akaike information criterion, *BIC* Bayesian information criterion. Sex is coded male = 0, female = 1

*p <.05; **p <.01; ***p <.001

**Table 4:** Hierarchical regression results for Kinsey scale, predicted from digit ratio (*N* = 315)

Variable	Model 1			Model 2			Model 3			Model 4		
	*B (SE)*	*95% CI*	*β*	*B (SE)*	*95% CI*	*β*	*B (SE)*	*95% CI*	*β*	*B (SE)*	*95% CI*	*β*
Constant	1.76*** (0.19)	1.39; 2.13		3.14 (2.86)	−2.49; 8.77		−9.40* (4.69)	−18.64; −0.17		−13.23 (11.55)	−35.98; 9.48	
SC1	0.07 (0.25)	−0.41; 0.56	0.02	0.08 (0.24)	−0.40; 0.55	0.21	0.10 (0.24)	−0.37; 0.56	0.03	10.72 (16.62)	−21.99; 43.42	3.01
SC2	−0.31 (0.25)	−0.79; 0.18	−0.09	−0.13 (0.24)	−0.61; 0.35	−0.04	−0.16 (0.24)	−0.63; 0.32	−0.04	3.10 (12.91)	−22.31; 28.51	0.87
Sex				0.86*** (0.21)	0.44; 1.28	0.23	20.55*** (5.90)	8.94; 32.16	5.57	26.62* (13.50)	0.05; 53.19	7.22
Digit ratio (DR)				−2.09 (2.97)	−7.93; 3.75	−0.04	10.96* (4.88)	1.36; 20.55	0.21	14.96 (12.04)	−8.72; 38.64	0.28
Sex x DR							−20.37*** (6.10)	−32.37; −8.37	−5.40	−26.65 (14.01)	−54.22; 0.92	−7.07
SC1 x Sex										0.37 (16.05)	−31.21; 31.96	0.09
SC2 x Sex										−18.37 (18.85)	−55.46; 18.72	−4.82
SC1 x DR										−3.39 (13.44)	−29.84; 23.06	−0.93
SC2 x DR										−11.08 (17.33)	−45.18; 23.02	−3.04
SC1 x Sex x DR										19.01 (19.57)	−19.50; 57.52	4.88
SC2 x Sex x DR										−0.34 (16.62)	−33.04; 32.36	−0.08
*R²*	0.010			0.059			0.092			0.099		
*ΔR²*	0.010			0.050***			0.033***			0.007		
*AIC*	1237.56			1225.26			1216.10			1225.63		
*BIC*	1252.46			1247.78			1242.36			1274.41		

*SC1* study contrast 1 (Study 2 [1] versus Study 1 [0]), *SC2* study contrast 2 (Study 3 [1] versus Study 1 [0]), *DR* digit ratio, *AIC* Akaike information criterion, *BIC* Bayesian information criterion. Sex is coded male = 0, female = 1

*p <.05; **p <.01; ***p <.001

**Table 5:** Hierarchical regression results for directional attraction, predicted from ulna-fibula ratio (UFR; *N* = 369)

Variable	Model 1			Model 2			Model 3			Model 4		
	*B (SE)*	*95% CI*	*β*	*B (SE)*	*95% CI*	*β*	*B (SE)*	*95% CI*	*β*	*B (SE)*	*95% CI*	*β*
Constant	0.90** (0.30)	0.31; 1.49		−0.39 (1.59)	−3.52; 2.75		2.57 (2.46)	−2.28; 7.42		13.38 (7.01)	−0.40; 27.16	
SC1	−0.18 (0.37)	−0.91; 0.55	−0.03	−0.08 (0.22)	−0.51; 0.35	−0.01	−0.11 (0.22)	−0.54; 0.33	−0.02	−14.23 (9.13)	−32.18; 3.73	−2.49
SC2	−1.03* (0.40)	−1.80; −0.25	−0.17	−0.00 (0.24)	−0.46; 0.46	−0.00	0.01 (0.23)	−0.45; 0.47	0.00	−11.80 (7.60)	−26.74; 3.15	−1.94
Sex				4.98*** (0.19)	4.60; 5.36	0.81	0.03 (3.15)	−6.17; 6.23	0.01	−10.86 (7.90)	−26.40; 4.67	−1.77
UFR				−3.75 (2.31)	−8.29; 0.79	−0.05	−8.07* (3.59)	−15.13; −1.01	−0.11	−23.79* (10.22)	−43.89; −3.69	−0.32
Sex x UFR							7.30 (4.65)	−1.84; 16.44	0.80	23.12* (11.58)	0.34; 45.90	2.54
SC1 x Sex										14.37 (10.46)	−6.20; 34.94	2.38
SC2 x Sex										11.14 (9.08)	−6.72; 29.00	1.50
SC1 x UFR										20.68 (13.31)	−5.49; 46.86	2.47
SC2 x UFR										17.03 (11.10)	−4.79; 38.86	1.88
SC1 x Sex x UFR										−21.07 (15.31)	−51.18; 9.03	−2.38
SC2 x Sex x UFR										−15.89 (13.39)	−42.22; 10.44	−1.41
*R²*	0.023			0.669			0.671			0.674		
*ΔR²*	0.023*			0.645***			0.002			0.003		
*AIC*	1816.65			1421.78			1421.28			1429.79		
*BIC*	1832.29			1445.24			1448.65			1480.63		

*SC1* study contrast 1 (Study 2 [1] versus Study 1 [0]), *SC2* study contrast 2 (Study 3 [1] versus Study 1 [0]), *UFR* ulna-fibula ratio, *AIC* Akaike information criterion, *BIC* Bayesian information criterion. Sex is coded male = 0, female = 1

*p <.05; **p <.01; ***p <.001

**Table 6:** Hierarchical regression results for Kinsey scale, predicted from ulna-fibula ratio (UFR;* N* = 369)

Variable	Model 1			Model 2			Model 3			Model 4		
	*B (SE)*	*95% CI*	*β*	*B (SE)*	*95% CI*	*β*	*B (SE)*	*95% CI*	*β*	*B (SE)*	*95% CI*	*β*
Constant	1.73*** (0.18)	1.38; 2.08		3.11 (1.60)	−0.03; 6.24		6.70** (2.46)	1.86; 11.54		17.38* (7.01)	3.61; 31.16	
SC1	0.07 (0.22)	−0.37; 0.51	0.02	0.11 (0.22)	−0.32; 0.55	0.03	0.08 (0.22)	−0.35; 0.51	0.02	−14.23 (9.13)	−32.18; 3.73	−4.16
SC2	−0.28* (0.24)	−0.74; 0.19	−0.08	−0.11 (0.24)	−0.57; 0.35	−0.03	−0.10 (0.23)	−0.56; 0.36	−0.03	−11.80 (7.60)	−26.74; 3.15	−3.25
Sex				0.86*** (0.19)	0.48; 1.24	0.24	−5.14 (3.15)	−11.34; 1.05	−1.40	−15.91* (7.90)	−31.44; −0.37	−4.33
UFR				−3.03 (2.31)	−7.58; 1.52	−0.07	−8.28* (3.59)	−15.34; −1.23	−0.19	−23.79* (10.22)	−43.89; −3.69	−0.54
Sex x UFR							8.87 (4.64)	−0.26; 18.00	1.63	24.46* (11.58)	1.68; 47.24	4.49
SC1 x Sex										14.08 (10.46)	−6.49; 34.65	3.90
SC2 x Sex										12.45 (9.08)	−5.41; 30.31	2.81
SC1 x UFR										20.68 (13.31)	−5.49; 46.86	4.13
SC2 x UFR										17.03 (11.10)	−4.79; 38.86	3.14
SC1 x Sex x UFR										−20.29 (15.31)	−50.39; 9.81	−3.83
SC2 x Sex x UFR										−18.18 (13.39)	−44.51; 8.15	−2.70
*R²*	0.008			0.071			0.080			0.088		
*ΔR²*	0.008			0.063***			0.009			0.008		
*AIC*	1442.69			1422.59			1420.90			1429.79		
*BIC*	1458.34			1446.05			1448.28			1480.63		

*SC1* study contrast 1 (Study 2 [1] versus Study 1 [0]), *SC2* study contrast 2 (Study 3 [1] versus Study 1 [0]), *UFR* ulna-fibula ratio, *AIC* Akaike information criterion, *BIC* Bayesian information criterion. Sex is coded male = 0, female = 1

*p <.05; **p <.01; ***p <.001

### Design

With regard to our hypothesis tests, our study had a correlational design, with digit ratio and UFR serving as predictors, natal sex as moderator, and androphilia, gynephilia, and derived measures as criteria in correlation and regression analyses.

### Measures

#### *Digit Ratio*

Participants placed their hands on the platen of a Hewlett-Packard Scanjet G3010 scanner so that both hands and their creases were visible in detail. Scans had a 1699 × 2340 pixels format and a 200 dpi horizontal and vertical resolution. The lengths of the second and fourth digits were measured from the tip of the finger to the midpoint of the bottom crease, using Face/Palm 1.0.7 (Köllner et al., 2017). This has been shown to be a highly reliable method for determining digit length (Kemper & Schwerdtfeger, 2009) and also in our own laboratory (Schultheiss et al., 2019). Digit ratio was first calculated separately for each hand by dividing the length of the 2nd digit by the length of the 4th digit. Ratio scores were then averaged across hands to obtain the overall digit ratio measure we used in our analyses.

#### *Ulna-to-fibula Ratio*

Ulna and fibula lengths were measured on seated, dressed participants (but with shoes, jackets, if possible pullovers etcetera removed) using the same devices (caliper, precision: 0.02 mm; arm-length-measuring-apparatus, cf. Figure 1 in Köllner and Bleck 2020a, b) and the same method as described in Köllner and Bleck ([Bibr CR19][Bibr CR20]; cf. Köllner et al. 2022a, b). Long bone length was assessed on both sides of the body as the distance between reference points for the ulna (caput ulnae to olecranon) and the fibula (caput fibulae to malleolus lateralis). This method is highly reliable (interrater reliabilities ranging from 0.81 to 0.99; Köllner and Bleck 2020a, b). UFR was calculated by dividing ulna length averaged across both arms by fibula length averaged across both legs.

**Fig. 1:**
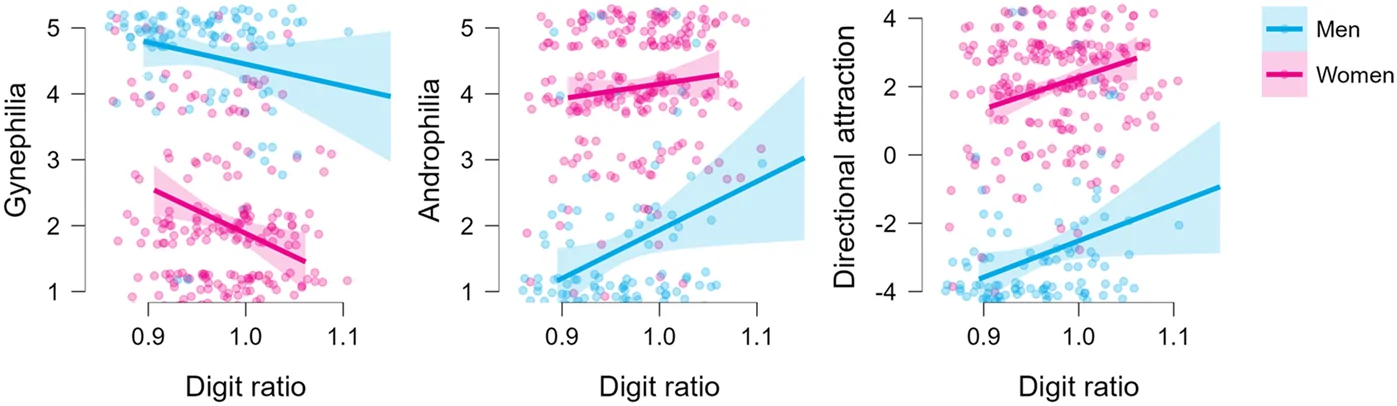
Scatter plots with linear regression lines (*95% CI*) for the association between digit ratio and gynephilia, androphilia, and directional attraction (androphilia minus gynephilia) in women (pink) and men (blue)

#### *Androphilia and Gynephilia*

Androphilia and gynephilia were assessed, respectively, with the items “How much do you feel sexually attracted to men [German: *Wie sehr fühlen Sie sich sexuell von Männern angezogen?*]” and “How much do you feel sexually attracted by women? [*Wie sehr fühlen Sie sich sexuell von Frauen angezogen?*]”. For both items, the response scale was 1 (not at all [*gar nicht*]), 2 (somewhat [*etwas*]), 3 (medium [*mittel*]), 4 (considerably [*stark*]), and 5 (very strongly [*sehr stark*]).

From these two scales, we additionally derived a Kinsey-type measure for comparative purposes with the following formulae:

Women: 4 + (gynephilia – androphilia).

Men: 4 + (androphilia - gynephilia).

The resulting scores could range from 0 (exclusively heterosexual attraction) to 8 (exclusively homosexual attraction), with scores from 1 to 7 representing increasing admixtures of homosexual attraction to a heterosexual attraction. The midscale point of 4 represented equal attraction to both genders.

### Open Science

All data and analyses are accessible on https://osf.io/682sg/.

## Results

Table 1 provides an overview of descriptive data for all measures, presented separately for women and men. Women scored considerably higher on androphilia than men and men scored considerably higher on gynephilia than women (see Supplement for histograms of these variables by sex). Consistent with this pattern, women also scored higher on the directional attraction measure than men. We also observed that women reported to be slightly less heterosexual on the Kinsey scale than men. Results for bone-marker measures were in the expected direction, too, with women having higher digit ratios and lower UFR values than men.

Sex composition differed significantly between studies, *χ²*(2) = 15.26, *p* <.001. A MANOVA testing for between-study effects on androphilia, gynephilia, digit ratio and UFR while simultaneously controlling for sex yielded a significant main effect of study, *F*(2, 308) = 6.14, Wilk’s *Λ* = 0.856, *p* <.001. Follow-up ANOVAs, again controlling for sex, showed that the overall effect was due to significant between-study differences in androphilia, gynephilia, and UFR, *p*s < 0.01, but not in digit ratio, *p* =.77. We therefore controlled for study in all subsequent analyses. Moreover, we also tested for possible interaction effects involving study in all focal analyses (see Tables 4, 5 and 6).

Gynephilia and androphilia were negatively correlated, associated with bone markers in opposite directions, and associations did not vary considerably by sex (see Table 2)^3^. Given this pattern of findings, we deemed the derivation of a measure of *directional attraction* justified, as it allowed us to simplify and condense result patterns. Directional attraction was calculated by subtracting gynephilia from androphilia. The resulting scale represents exclusive attraction to men at its highest possible score (4) and exclusive attraction to women at its lowest possible score (−4). Less extreme scores could result from either varying admixtures of androphilia and gynephilia or a combination of the minimum score on one scale and a lower-than-maximum score on the other. Zero represents the score at which attraction to men and women is exactly balanced, because both scales were endorsed at an equal level (e.g., both at 5 or both at 1 or any exact match in between).

To systematically predict sexual attraction measures in the overall sample, we built hierarchical linear regression models with Study representing Model 1, adding Sex and Digit Ratio in Model 2 to test the independent contributions of these variables to the prediction, the Sex x Digit Ratio interaction in Model 3 to test for sex-moderated associations between digit ratio and sexual attraction, and finally, in Model 4, all terms required to test the full model leading up to the Study x Sex x Digit Ratio interaction. As shown in Table 3, Model 2 provides the best fit according to Akaike’s and Bayesian information criteria (AIC and BIC) and suggests that sex and digit ratio represent independent significant predictors of directional sexual attraction. Higher digit ratio predicted more androphilia and correspondingly less gynephilia in both men and women, although – due the simultaneous main effect of sex – at different levels. Separate analyses conducted for gynephilia and androphilia (see Supplement) indicate that this pattern of findings derives in equal part from each of the measures constituting directional attraction (see Fig. 1 for an illustration of these findings). In contrast, Models 3 and 4 with their interaction effects do not add any significant predictive power to account for variations in sexual attraction, and their fit indices deteriorate relative to Model 3.

We repeated these analyses with the Kinsey scale substituting the directional attraction scale. As shown in Table 4, digit ratio failed to account for significant portions of variance in this criterion variable in Model 2, although sex was a significant predictor, with women scoring higher on the Kinsey scale than men. For the Kinsey scale, the best-fitting model was Model 3, which featured a significant Sex x Digit Ratio interaction. As suggested by the correlations reported in Table 2, this was due to a negative association between digit ratio and Kinsey scores in women and a positive one in men.

**Fig. 2:**
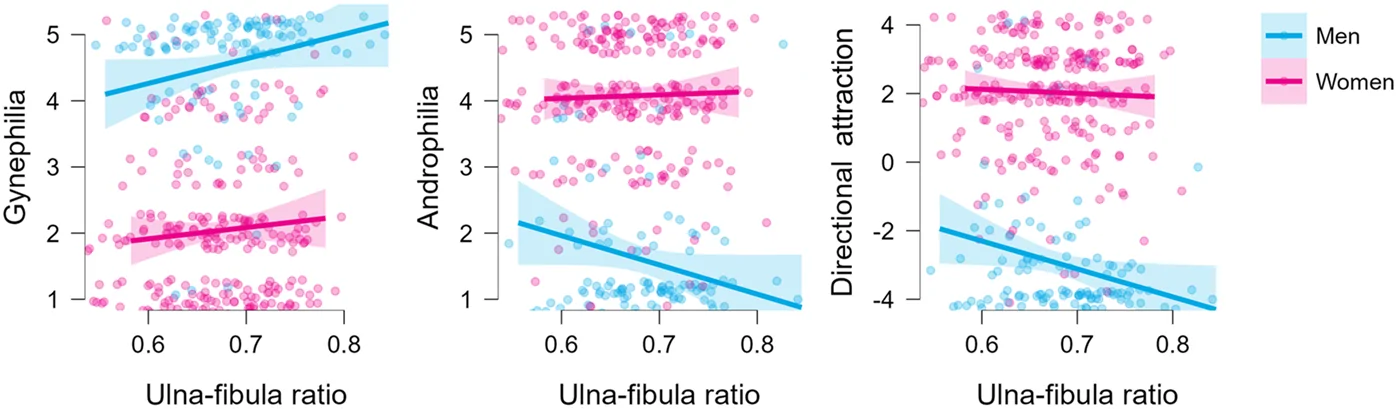
Scatter plots with linear regression lines (*95% CI*) for the association between ulna-fibula-ratio and gynephilia, androphilia, and directional attraction (androphilia minus gynephilia) in women (pink) and men (blue)

Exploratory analyses for UFR using the same hierarchical-regression approach for the prediction of directional attraction again indicated the best fit for Model 3. However, UFR was not a significant predictor of directional attraction. In this case, separate analyses for the disaggregated androphilia and gynephilia scales suggested a more subtle pattern that was obscured by the composite score (see Supplement). For gynephilia, UFR was a significant positive predictor in Model 3, suggesting that more male-typical UFR was associated with greater attraction to women in both sexes. In contrast, for androphilia Model 4 provided a slightly better fit by the AIC criterion (though not by the BIC criterion), and the Sex x UFR interaction term was significant, due to a positive association between UFR and androphilia in women and a negative one in men (both non-significant; see Table 2). Figure 2 illustrates these findings for directional attraction and separate attraction scales. In all analyses, the addition of the terms included in the full Sex x Study x UFR interaction effect did not improve model fit. Also, replacing the directional attraction criterion measure with the Kinsey scale did not result in a clearer overall picture (see Table 6). UFR was not substantially associated with digit ratio in the overall sample or in each sex (see Table 2).

## Discussion

We have analyzed associations between bone-marker measures of organizational hormone effects and allocentric (gynephilia, androphilia, plus directional attraction as a summary variable) and egocentric (Kinsey-type scale) measures of sexual orientation in a combined sample resulting from three separate studies. Our results were mostly in keeping with our hypotheses. As predicted and consistent with the extant research literature, women were higher in androphilia and lower in gynephilia than men, with effect sizes in the very large range (*d*s ≥ 2.40). On a Kinsey-type scale derived from androphilia and gynephilia measures, women also turned out to be slightly less heterosexually oriented than men, with a medium effect size. This finding is consistent with a secular trend towards less exclusively heterosexual orientations among women, but not among men (e.g., Massey et al., 2021; Rahman et al., 2020; Savin-Williams, 2016). Women also had higher digit ratios and lower ulna-fibula ratios (UFR) than men, with effect sizes in the medium range and thus comparable to earlier studies (e.g., Butovskaya et al. 2021; Köllner and Bleck 2020a, b; Köllner et al. 2022a, b).

Our measures thus performed as would be expected based on the extant literature and thus enabled us to test our main hypotheses, which focused on associations between measures of organizational hormone effects (prenatal: digit ratio; pubertal: UFR) and allocentric versus egocentric measures of sexual orientation. Turning to the latter first, we replicated the meta-analytically well-established finding of a negative association between digit ratio and homosexual orientation among women and a positive association between these measures among men, with effect sizes in both cases in the small-to-medium range. These findings suggest that more prenatal exposure to testosterone than estradiol is associated with a more homosexual orientation among women and a more heterosexual orientation in men and, as Grimbos et al. (2010) have argued, that sex is a moderator of the association between prenatal hormones and sexual attraction. Our effect sizes were slightly larger than those reported in the literature -- Swift-Gallant et al.’s (2025) effect sizes, converted to Pearson’s *r*, ranged from − 0.09 to − 0.10 for women (our estimate was − 0.18) and from 0.08 to 0.13 for men (our estimate was 0.21). The difference may be due to our comparatively smaller overall sample (smaller samples may inflate effect size estimates; see Loken & Gelman, 2017; Schönbrodt & Perugini, 2013) and our re-construction of a Kinsey-type measure from allocentric measures of sexual orientation, which may differ in their diagnostic properties from straightforward self-report Kinsey-type scales.

However, with regard to the difference between egocentric and allocentric measures of sexual orientation, our results also illustrate the problem with the former by providing greater clarity through the latter. As just one glance at Fig. 1 reveals, digit ratio was positively associated with more attraction to men and less to women. This finding held similarly for women and men, for the directional difference score and for the androphilia and gynephilia measures the difference score is based on. Thus, despite large differences in gynephilia and androphilia and also typical differences in digit ratio between women and men, relatively more exposure to estradiol and relatively less exposure to testosterone prenatally predisposed *all* tested individuals to feel more attracted to men and less attracted to women. Sex was decidedly *not* a moderator of the association between prenatal steroid exposure and sexual attraction measures. This was also confirmed by our regression analyses. For allocentric sexual attraction measures, the simpler main effects model including both sex and digit ratio performed best, whereas for the egocentric Kinsey-type scale, the more complex sex-as-moderator model provided the best fit. Thus, compared to egocentric measures, allocentric measures of sexual attraction are not only more transparent, they also provide simpler and therefore more robust predictive models. Overall, then, we were able to confirm our third and fourth hypotheses, which we had deduced from the meta-analytical findings based on egocentric Kinsey measures of sexual orientation, and to clarify the nature of the association between a measure of prenatal hormone exposure and measures of sexual attraction.

Our exploratory analyses for UFR indicated that a more male-typical pattern, reflecting higher testosterone and perhaps also less estradiol during puberty, was associated with relatively less sexual attraction to men and more sexual attraction to women. However, this finding was restricted to men; in women, associations between allocentric sexual orientation measures and UFR were consistently small and confidence intervals included zero. We did not observe systematic associations between digit ratio and UFR within and across sexes, a finding that supports the idea that developmentally independent processes shape these bone markers measures. Further research is needed to examine the robustness of the associations involving UFR we observed in the present study and to explore the additive and interactive effects of digit ratio and UFR on measures of sexual attraction.

## Limitations

One limitation of our study is the lack of a generic, self-report Kinsey scale, such as it was used in many studies entering the meta-analysis by Swift-Gallant et al. (2025). We used a derived scale, calculated on the basis of scale scores for androphilia, gynephilia, and participants’ natal sex. The correlation of our derived Kinsey-type scale with generic Kinsey scales is unknown, although we suggest that one can plausibly expect it to be in the high positive range (> 0.50).

Another limitation is our reliance on single-item measures of androphilia and gynephilia. We asked very directly about participants’ *sexual* attraction to women or men. However, other researchers have used measures of androphilia and gynephilia that also included items assessing romantic and other aspects of attraction (e.g., Freund & Blanchard, 2011; Hale et al., 2019). Using multiple items per scale not only allows the calculation of an estimate of reliability in terms of internal scale consistency but may also capture a broader concept of sexual attraction. Moreover, it would be worthwhile to test whether, as we assume, our sexual-attraction findings also extend to the frequency of sexual behavior with female and male partners.

A third limitation is the lack of reliability data for our morphological measures, digit ratio and UFR. However, given the documented high precision with which our lab has assessed these variables in past published studies and our thorough training of the individuals who carried out the measurements, we are confident that the reliability of the measurements we used in the present research was sufficient to test our hypotheses.

Another limitation associated with our morphological measures is much more difficult to resolve and brings us back to our introductory discussion of the problems with difference scores. Digit ratio, and presumably also UFR (see Köllner et al. 2022a, b), represent measures of the relative contribution of the organizational effects of testosterone and estradiol, with these hormones “pulling” the measure in opposite directions in each case. Thus, these morphological markers represent reflections of a difference effect of two gonadal steroids, and it remains unclear in our research – like in many other studies – whether both hormones contribute equally to the net effect of each ratio score or one is more relevant than the other. Unless some additional tell-tale markers of specific estrogenic or androgenic organizational effects during development are identified that can help further qualify the information that can be gleaned from bone marker measures, it may be impossible to resolve this issue except by monitoring endogenous hormone levels directly and continually during sensitive windows of development.

Finally, at first blush our use of a difference measure -- directional attraction -- may appear to contradict our arguments against such measures laid out in the introduction. However, our argument was directed at a-priori difference measures. In contrast, we derived our directional attraction difference measure only after ascertaining that (1) the scales constituting it – gynephilia and androphilia – both captured a relevant association with an outcome measure and (2) that this effect went in opposite directions. Thus, directional attraction was derived a posteriori, to efficiently summarize and illustrate findings that were already present at the level of its constituting measures, and we transparently also reported all associations for gynephilia and androphilia separately in the tables. We therefore deem this approach to be more justifiable than one that uses a difference measure from the get-go, without making the empirical basis for creating it transparent and compelling.

## Conclusion

Concepts such as heterosexuality, homosexuality, bisexuality, and so on may be useful ways for individuals to identify their perceived sexual orientation. But they remain politically fraught categories (see Bailey et al., 2016) and, as we hope to have shown here, they are also methodologically problematic: They represent a dimension-conflating, egocentric approach toward measuring sexual attraction. Sexual attractions toward women and men are better assessed as separate dimensions and in an allocentric manner. By using the latter approach through the assessment of self-reported gynephilia and androphilia, we were able to show consistent and sex-independent associations with morphological markers of organizational hormone effects. Thus, a simple switch at the level of measurement from egocentric to allocentric measurement of sexual orientation that retains all options for reclassifying individuals as homo- or heterosexual (if one desires to do so) may help to clarify some important developmental roots of sexual desire and holds considerable promise for research on other causes, correlates, and consequences of sexual orientation.

## Supplementary Information

Below is the link to the electronic supplementary material.

Supplementary Material 1 (DOCX 132 KB)

## Data Availability

All data and analyses are accessible on [https://osf.io/682sg/].

## Footnotes

1 One reviewer pointed out that in addition to assessing sexual orientation from an allocentric point of view, it is equally important to measure and conceptualize gender and sex independently in such research.

2 Please note that we are referring with “men” and “women” to the natal sex of our participants in our methods and results sections. Because we also queried participants about their gender, we know that in the total sample two natal males self-identified as women and one as non-binary and that four natal women self-identified as men and two as non-binary.

3 As one reviewer pointed out, although the correlation between androphilia and gynephilia was negative in both sexes, Table 2 shows it to be considerably greater in men than in women, with the difference between the correlation coefficients being significant, *z* = 3.85, *p* <.001. Thus, although the negative correlation and practical considerations of the present research justify the post-hoc derivation of a difference measure of sexual orientation, keeping sexual orientation measures separate may be particularly relevant in future research aiming to better understand the greater variation of female sexual orientation.
