# Correction: Deconstructing Kinsey's Scale: Digit Ratio Correlates Negatively with Gynephilia and Positively with Androphilia in Both Sexes

**DOI:** 10.1007/s12110-026-09522-3

**Published:** 2026-04-29

**Authors:** Oliver C. Schultheiss, David Gebhardt, Elisabeth Meier, Sophie Steck, Monika Wulf, Paul Compensis, Martin G. Köllner

**Affiliations:** 1 Friedrich-Alexander University, Erlangen, Germany; 2 SRH University of Applied Sciences Heidelberg, Fürth, Germany

**Correction to: Human Nature**

10.1007/s12110-026-09518-z

In this article, several errors introduced during the production process were identified. These errors do not affect the scientific content, analyses, or conclusions of the paper. The corrections are detailed below.

1. Formatting of Statistical Abbreviations
  In several instances throughout the main text and tables, statistical abbreviations (e.g., *M*, *SD*, *d*, 95% *CI*, *B*, *SE*) were not italicized in accordance with APA style guidelines. These formatting errors have been corrected.
2. Reference List Order
  The reference list contained ordering errors introduced during production. Specifically, the reference beginning with “Agmo 2007” was misplaced and appeared out of alphabetical sequence. The reference list has been corrected to ensure all entries are properly ordered alphabetically.
3. Figure Axis Labels
  In Figs. 1 and 2, the y-axis label of the rightmost panels was incorrectly given as “directional orientation.” The correct label is “directional attraction” and should appear below.

**Fig. 1:**
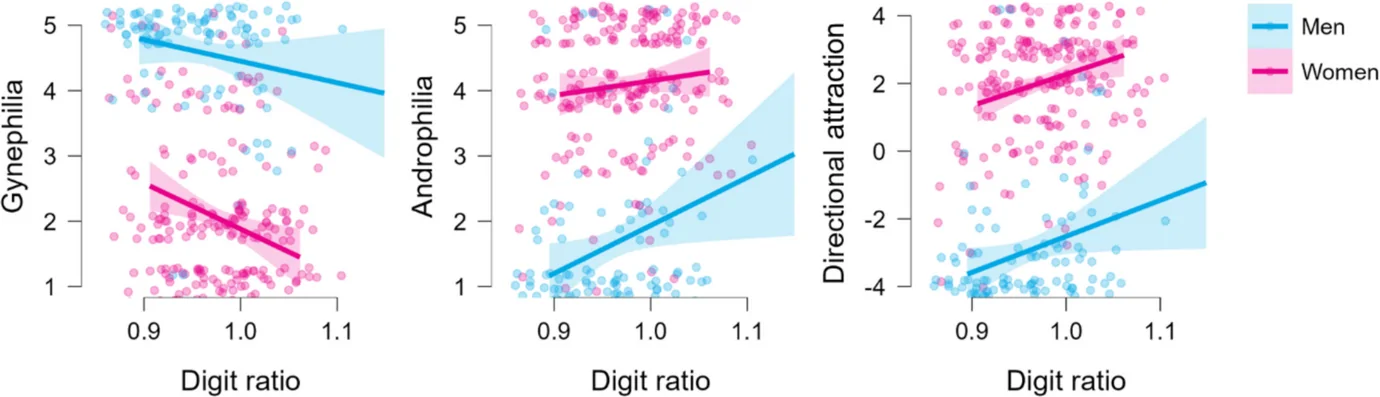
Scatter plots with linear regression lines (*95% CI*) for the association between digit ratio and gynephilia, androphilia, and directional attraction (androphilia minus gynephilia) in women (pink) and men (blue)

**Fig. 2:**
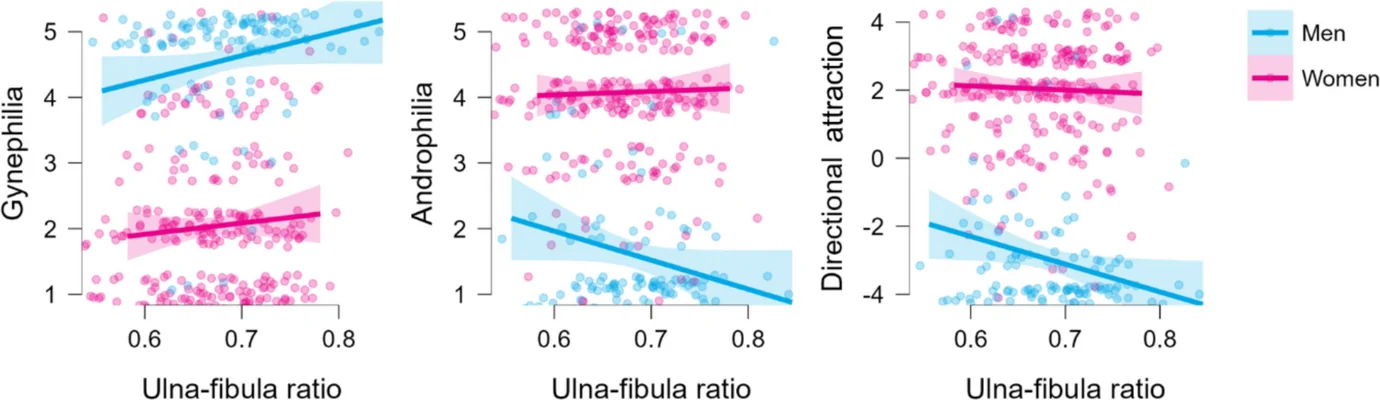
Scatter plots with linear regression lines (*95% CI*) for the association between ulna-fibula-ratio and gynephilia, androphilia, and directional attraction (androphilia minus gynephilia) in women (pink) and men (blue)

The original article has been corrected.

