# Supplementary material for: Deconstructing Kinsey's Scale: Digit Ratio Correlates Negatively with Gynephilia and Positively with Androphilia in Both Sexes
Source: Hum Nat. 2026 Apr 15;37(2):150–71. doi: 10.1007/s12110-026-09518-z (PMC13506557; doi:10.1007/s12110-026-09518-z)
Supplement: Supplementary file 1 [file 12110_2026_9518_MOESM1_ESM.docx]

Running head: DECONSTRUCTING KINSEY SUPPLEMENT

**Deconstructing Kinsey’s scale:**

**Digit ratio correlates negatively with gynephilia and positively with androphilia in both sexes**

**Supplement**

Oliver C. Schultheiss^1^

David Gebhardt^1^

Elisabeth Meier^1^

Sophie Steck^1^

Monika Wulf^1^

Paul Compensis^1^

Martin G. Köllner^1^

^1^Friedrich-Alexander University, Erlangen, Germany

^2^SRH University of Applied Sciences, Heidelberg, Germany

**Figure 1**

*Histograms, by sex, for gynephilia, androphilia, directional orientation (ORIENTDIFFSCALE) and Kinsey scale (PSEUDOKINSEY)*

**Table 1**

*Hierarchical regression results for gynephilia, predicted from digit ratio (N = 315)*

| Variable | Model 1 | | | Model 2 | | | Model 3 | | | Model 4 | | |
| --- | --- | --- | --- | --- | --- | --- | --- | --- | --- | --- | --- | --- |
|  | *B (SE)* | *95% CI* | *β* | *B (SE)* | *95% CI* | *β* | *B (SE)* | *95% CI* | *β* | *B (SE)* | *95% CI* | *β* |
| Constant | 2.71*** (0.09) | 2.38; 3.05 |  | 10.03*** (1.80) | 6.48; 13.58 |  | 7.95** (3.01) | 2.03; 13.87 |  | 11.72 (7.39) | -2.82; 26.25 |  |
| SC1 | -0.19 (0.22) | -0.63; 0.25 | -0.06 | -0.17 (1.51) | -0.47; 0.13 | -0.05 | -0.17 (0.15) | -0.47; 0.13 | -0.05 | -2.57 (10.63) | -23.48; 18.35 | -0.78 |
| SC2 | 0.54* (0.22) | 0.19; 0.98 | 0.16 | 0.02 (1.51) | -0.28; 0.32 | 0.01 | 0.02 (0.15) | -0.29; 0.32 | 0.01 | -4.48 (8.26) | -20.74; 11.77 | -1.36 |
| Sex |  |  |  | -2.42*** (0.13) | -2.69; -2.16 | -0.71 | 0.85 (3.78) | -6.59; 8.29 | 0.25 | -7.21 (8.64) | -24.20; 9.79 | -2.11 |
| Digit ratio (DR) |  |  |  | -5.63** (1.87) | -9.32; -1.95 | -0.12 | -3.47 (3.13) | -9.62; 2.69 | -0.07 | -7.53 (7.70) | -22.67; 7.62 | -0.15 |
| Sex x DR |  |  |  |  |  |  | -3.38 (3.91) | -11.08; 4.31 | -0.97 | 5.11 (8.96) | -12.52; 22.74 | 1.46 |
| SC1 x Sex |  |  |  |  |  |  |  |  |  | 11.15 (10.23) | -9.05; 31.36 | 2.83 |
| SC2 x Sex |  |  |  |  |  |  |  |  |  | 7.78 (12.01) | -15.94; 31.50 | 2.20 |
| SC1 x DR |  |  |  |  |  |  |  |  |  | 4.93 (8.60) | -11.99; 21.85 | 1.46 |
| SC2 x DR |  |  |  |  |  |  |  |  |  | 2.50 (11.08) | -19.31; 24.30 | 0.74 |
| SC1 x Sex x DR |  |  |  |  |  |  |  |  |  | -8.01 (12.52) | -32.65; 16.62 | -2.22 |
| SC2 x Sex x DR |  |  |  |  |  |  |  |  |  | -11.89 (10.63) | -32.80; 9.03 | -2.94 |
| *R²* | .041 |  |  | 0.564 |  |  | 0.565 |  |  | 0.571 |  |  |
| *ΔR²* | .041*** |  |  | 0.523*** |  |  | 0.001 |  |  | 0.005 |  |  |
| *AIC* | 1179.20 |  |  | 934.63 |  |  | 935.87 |  |  | 944.09 |  |  |
| *BIC* | 1194.21 |  |  | 957.14 |  |  | 962.13 |  |  | 992.87 |  |  |

*Note.* SC1 = study contrast 1 (Study 2 [1] versus Study 1 [0]); SC2 = study contrast 2 (Study 3 [1] versus Study 1 [0]); DR = digit ratio; AIC = Akaike information criterion; BIC = Bayesian information criterion. Sex is coded male = 0, female = 1.

**p* < .05; ***p* < .01; ****p* < .001.

**Table 2**

*Hierarchical regression results for androphilia, predicted from digit ratio (N = 315)*

| Variable | Model 1 | | | Model 2 | | | Model 3 | | | Model 4 | | |
| --- | --- | --- | --- | --- | --- | --- | --- | --- | --- | --- | --- | --- |
|  | *B (SE)* | *95% CI* | *β* | *B (SE)* | *95% CI* | *β* | *B (SE)* | *95% CI* | *β* | *B (SE)* | *95% CI* | *β* |
| Constant | 3.57*** (0.17) | 3.25; 3.90 |  | -2.28 (1.73) | -5.69; 1.13 |  | -5.13 (2.89) | -10.82; 0.56 |  | -5.53 (7.04) | -19.38; 8.31 |  |
| SC1 | -0.21 (0.22) | -0.64; 0.22 | -0.07 | -0.22 (0.15) | -0.51; 0.06 | -0.07 | -0.22 (0.15) | -0.51; 0.07 | -0.07 | 8.15 (10.12) | -11.77; 28.07 | 2.57 |
| SC2 | -0.45* (0.22) | -0.88; -0.02 | -0.14 | 0.06 (0.15) | -0.23; 0.35 | 0.02 | 0.06 (0.15) | -0.23; 0.35 | 0.02 | -1.38 (7.87) | -16.86; 14.10 | -0.44 |
| Sex |  |  |  | 2.43*** (0.13) | 2.17; 2.68 | 0.74 | 6.90 (3.63) | -0.25; 14.05 | 2.10 | 0.68 (8.23) | -15.51; 16.86 | 0.21 |
| Digit ratio (DR) |  |  |  | 4.12* (1.80) | 0.58; 7.66 | 0.09 | 7.09* (3.00) | 1.18; 12.99 | 0.15 | 7.44 (7.33) | -6.99; 21.86 | 0.16 |
| Sex x DR |  |  |  |  |  |  | -4.63 (3.75) | -12.01; 2.76 | -1.38 | 1.84 (8.53) | -14.96; 18.63 | 0.55 |
| SC1 x Sex |  |  |  |  |  |  |  |  |  | 4.58 (9.78) | -14.66; 23.82 | 1.21 |
| SC2 x Sex |  |  |  |  |  |  |  |  |  | 4.72 (11.48) | -17.87; 27.31 | 1.39 |
| SC1 x DR |  |  |  |  |  |  |  |  |  | 1.54 (8.19) | -14.57; 17.65 | 0.47 |
| SC2 x DR |  |  |  |  |  |  |  |  |  | -8.59 (10.56) | -29.35; 12.19 | -2.64 |
| SC1 x Sex x DR |  |  |  |  |  |  |  |  |  | -4.86 (11.92) | -28.32; 18.60 | -1.40 |
| SC2 x Sex x DR |  |  |  |  |  |  |  |  |  | -4.77 (10.12) | -24.69; 15.15 | -1.23 |
| *R²* | .014 |  |  | 0.565 |  |  | 0.567 |  |  | 0.580 |  |  |
| *ΔR²* | .014 |  |  | 0.551*** |  |  | 0.002 |  |  | 0.012 |  |  |
| *AIC* | 1163.88 |  |  | 909.89 |  |  | 910.35 |  |  | 913.35 |  |  |
| *BIC* | 1178.89 |  |  | 932.41 |  |  | 936.62 |  |  | 962.13 |  |  |

*Note.* SC1 = study contrast 1 (Study 2 [1] versus Study 1 [0]); SC2 = study contrast 2 (Study 3 [1] versus Study 1 [0]); DR = digit ratio; AIC = Akaike information criterion; BIC = Bayesian information criterion. Sex is coded male = 0, female = 1.

**p* < .05; ***p* < .01; ****p* < .001.

**Table 4**

*Hierarchical regression results for gynephilia, predicted from ulna-fibula ratio (N = 369)*

| Variable | Model 1 | | | Model 2 | | | Model 3 | | | Model 4 | | |
| --- | --- | --- | --- | --- | --- | --- | --- | --- | --- | --- | --- | --- |
|  | *B (SE)* | *95% CI* | *β* | *B (SE)* | *95% CI* | *β* | *B (SE)* | *95% CI* | *β* | *B (SE)* | *95% CI* | *β* |
| Constant | 2.70*** (0.17) | 2.37; 3.02 |  | 2.53* (1.02) | 0.53; 4.54 |  | 1.98 (1.58) | -1.13; 5.08 |  | -1.89 (4.49) | -10.72; 6.95 |  |
| SC1 | -0.12 (0.21) | -0.52; 0.29 | -0.04 | -0.18 (0.14) | -0.46; 0.09 | -0.06 | -0.18 (0.14) | -0.45; 0.10 | -0.06 | 4.77 (5.86) | -6.74; 16.29 | -2.49 |
| SC2 | 0.54* (0.22) | 0.11; 0.97 | 0.16 | 0.04 (0.15) | -0.26; 0.33 | 0.01 | 0.04 (0.15) | -0.26; 0.44 | 0.01 | 4.22 (4.87) | -5.36; 13.81 | -1.94 |
| Sex |  |  |  | -2.47*** (0.12) | -2.71; -2.23 | -0.73 | -1.54 (2.02) | -5.51; 2.43 | -0.45 | 2.40 (5.07) | -7.56; 12.36 | -1.77 |
| UFR |  |  |  | 3.05* (1.48) | 0.15; 5.95 | 0.07 | 3.87 (2.30) | -0.65; 8.39 | 0.09 | 9.29 (6.55) | -3.60; 22.18 | -0.32 |
| Sex x UFR |  |  |  |  |  |  | -1.38 (2.98) | -7.23; 4.48 | -0.27 | -6.85 (7.43) | -21.46; 7.76 | 2.54 |
| SC1 x Sex |  |  |  |  |  |  |  |  |  | -4.77 (6.71) | -17.96; 8.43 | 2.38 |
| SC2 x Sex |  |  |  |  |  |  |  |  |  | -4.04 (5.82) | -15.50; 7.41 | 1.50 |
| SC1 x UFR |  |  |  |  |  |  |  |  |  | -7.09 (8.54) | -23.87; 9.70 | 2.47 |
| SC2 x UFR |  |  |  |  |  |  |  |  |  | -5.76 (7.12) | -19.76; 8.23 | 1.88 |
| SC1 x Sex x UFR |  |  |  |  |  |  |  |  |  | 6.77 (9.82) | -12.53; 26.08 | -2.38 |
| SC2 x Sex x UFR |  |  |  |  |  |  |  |  |  | 5.35 (8.59) | -11.53; 22.24 | -1.41 |
| *R²* | .034 |  |  | 0.561 |  |  | 0.561 |  |  | 0.564 |  |  |
| *ΔR²* | .034** |  |  | 0.527*** |  |  | 0.000 |  |  | 0.003 |  |  |
| *AIC* | 1377.35 |  |  | 1090.49 |  |  | 1092.27 |  |  | 1101.90 |  |  |
| *BIC* | 1392.99 |  |  | 1113.95 |  |  | 1119.65 |  |  | 1152.74 |  |  |

*Note.* SC1 = study contrast 1 (Study 2 [1] versus Study 1 [0]); SC2 = study contrast 2 (Study 3 [1] versus Study 1 [0]); UFR = ulna-fibula ratio; AIC = Akaike information criterion; BIC = Bayesian information criterion. Sex is coded male = 0, female = 1.

**p* < .05; ***p* < .01; ****p* < .001.

**Table 4**

*Hierarchical regression results for androphilia, predicted from ulna-fibula ratio (N = 369)*

| Variable | Model 1 | | | Model 2 | | | Model 3 | | | Model 4 | | |
| --- | --- | --- | --- | --- | --- | --- | --- | --- | --- | --- | --- | --- |
|  | *B (SE)* | *95% CI* | *β* | *B (SE)* | *95% CI* | *β* | *B (SE)* | *95% CI* | *β* | *B (SE)* | *95% CI* | *β* |
| Constant | 3.60*** (0.16) | 3.28; 3.91 |  | 2.15* (0.97) | 0.24; 4.06 |  | 4.55** (1.50) | 1.61; 7.49 |  | 11.50** (4.25) | 3.14; 19.86 |  |
| SC1 | -0.30 (0.20) | -0.69; 0.10 | -0.10 | -0.26 (0.13) | -0.52; 0.00 | -0.08 | -0.28* (0.13) | -0.54; -0.02 | -0.09 | -9.46 (5.54) | -20.35; 1.44 | -3.08 |
| SC2 | -0.49* (0.21) | -0.91; -0.07 | -0.15 | 0.04 (0.14) | -0.25; 0.32 | 0.01 | 0.04 (0.14) | -0.24; 0.32 | 0.01 | -7.57 (4.61) | -16.64; 1.50 | -2.32 |
| Sex |  |  |  | 2.51*** (0.12) | 2.28; 2.74 | 0.76 | -1.50 (1.91) | -5.27; 2.26 | -0.46 | -8.46 (4.79) | -17.89; 0.96 | -2.57 |
| UFR |  |  |  | -0.70 (1.41) | -3.47; 2.08 | -0.02 | -4.21 (2.18) | -8.49; 0.08 | -0.11 | -14.50* (6.20) | -26.69; -2.30 | -0.36 |
| Sex x UFR |  |  |  |  |  |  | 5.93* (2.82) | 0.38; 11.47 | 1.21 | 16.27* (7.03) | 2.45; 30.09 | 3.33 |
| SC1 x Sex |  |  |  |  |  |  |  |  |  | 9.61 (6.35) | -2.87; 22.09 | 2.96 |
| SC2 x Sex |  |  |  |  |  |  |  |  |  | 7.10 (5.51) | -3.74; 17.93 | 1.79 |
| SC1 x UFR |  |  |  |  |  |  |  |  |  | 13.58 (8.08) | -2.28; 29.48 | 3.03 |
| SC2 x UFR |  |  |  |  |  |  |  |  |  | 11.27 (6.73) | -1.97; 24.51 | 2.31 |
| SC1 x Sex x UFR |  |  |  |  |  |  |  |  |  | -14.30 (9.29) | -32.56; 3.96 | -3.01 |
| SC2 x Sex x UFR |  |  |  |  |  |  |  |  |  | -10.54 (8.12) | -26.51; 5.44 | -1.74 |
| *R²* | .014 |  |  | 0.574 |  |  | 0.579 |  |  | 0.583 |  |  |
| *ΔR²* | .014 |  |  | 0.560*** |  |  | 0.005* |  |  | 0.004 |  |  |
| *AIC* | 1360.78 |  |  | 1055.37 |  |  | 1052.91 |  |  | 1061.03 |  |  |
| *BIC* | 1376.42 |  |  | 1078.84 |  |  | 1080.29 |  |  | 1111.86 |  |  |

*Note.* SC1 = study contrast 1 (Study 2 [1] versus Study 1 [0]); SC2 = study contrast 2 (Study 3 [1] versus Study 1 [0]); UFR = ulna-fibula ratio; AIC = Akaike information criterion; BIC = Bayesian information criterion. Sex is coded male = 0, female = 1.

**p* < .05; ***p* < .01; ****p* < .001.

***Figure 1.*** Scatter plots with linear regression lines for the association between digit ratio and directional attraction (androphilia minus gynephilia) in Studies 1 to 3

***Figure 2.*** Scatter plots with linear regression lines for the association between ulna-fibula-ratio and directional attraction (androphilia minus gynephilia) in Studies 1 to 3
